# Industrial cylinder liner defect detection using a transformer with a block division and mask mechanism

**DOI:** 10.1038/s41598-022-14971-8

**Published:** 2022-06-23

**Authors:** Qian Liu, Xiaohua Huang, Xiuyan Shao, Fei Hao

**Affiliations:** 1 School of Computer Engineering, Nanjing Institute of Technology, Nanjing, China; 2 School of Economic and Management, Southeast University, Nanjing, JiangSu China; 3 School of Mechanical Engineering, Nanjing Institute of Technology, Nanjing, JiangSu China; 4 Advanced Industrial Technology Research Institute, Nanjing Institute of Engineering, Nanjing, JiangSu China

**Keywords:** Computer science, Software, Mechanical engineering

## Abstract

In the field of artificial intelligence, a large number of promising tools, such as condition-based maintenance, are available for large internal combustion engines. The cylinder liner, which is a key engine component, is subject to defects due to the manufacturing process. In addition, the cylinder liner straightforwardly affects the usage and safety of the internal combustion engine. Currently, the detection of cylinder liner quality mainly depends on manual human detection. However, this type of detection is destructive, time-consuming, and expensive. In this paper, a new cylinder liner defect database is proposed. The goal of this research is to develop a nondestructive yet reliable method for quantifying the surface condition of the cylinder liner. For this purpose, we propose a transformer method with a block division and mask mechanism on our newly collected cylinder liner defect database to automatically detect defects. Specifically, we first use a local defect dataset to train the transformer network. With a hierarchical-level architecture and attention mechanism, multi-level and discriminative feature are obtained. Then, we combine the transformer network with the block division method to detect defects in 64 local regions, and merge their results for the high-resolution image. The block division method can be used to resolve the difficulty of the in detecting the small defect. Finally, we design a mask to suppress the influence of noise. All methods allow us to achieve higher accuracy results than state-of-the-art algorithms. Additionally, we show the baseline results on the new database.

## Introduction

Surface defects leads directly to quality problems of the product, additionally affect the chemical and physical properties of the product surface. The cylinder liner is a key component of the internal combustion engine. Therefore, the appearance of cylinder liner surface defects, such as cracks and sand holes, will result in quality and safety problems in the engine. As a result, manufacturers have proposed strict industrial requirements for guaranteeing the quality of cylinder liners during the production process. Currently, the detection of cylinder liner surface defect quality mainly relies on manual visual testing. However, manual visual testing cannot meet the production requirements in terms of work efficiency. In addition, this type of detection suffers from human factors, such as emotion and subjective experience. Moreover, some product defects are small in size and diverse in shape, and it is difficult for human eyes to observe these defects. Additionally, detection may be harmful to the testers’ health, Therefore, manual detection cannot meet the requirements of current mass industrial production.

The nondestructive testing (NDT) method is another way for manufacturers to inspect defects. From an industrial viewpoint, the purpose of NDT is to determine whether a material or part will satisfactorily perform its intended function. This method is mainly focuses on the many aspects of the quality and serviceability of materials and structures and incorporates all the technologies for process monitoring and the detection and measurement of significant properties. For example, Dong et al. used X-Ray to analyze the abnormalities in the area around the weld^1^. Hato et al. used a high-speed scanning laser observation system to nondestructively test each layer of GdBa2Cu3O7-x (GdBCO) ion-beam-assisted-deposition and pulsed-laser-deposition (IBAD-PLD) coated conductor^2^. In the last two decades, the rapid development of the machine vision-based detection algorithm has promoted the development of surface defect detection technology. It has also promoted the computer vision platform as one method of NDT. Compared with manual detection and NDT with a scanning laser, a machine vision-based platform not only improves the efficiency of detection, but is also economic and flexible for manufacturers. For example, Liu et al. proposed an improved Particle Swarm Optimization Support Vector Machine (PSO-SVM) based on imaging technology to detect the defects of a vortex^3^. Although traditional machine vision algorithms exhibit efficiency in defect detection, professional knowledge is required. Therefore, it is desirable to directly obtain high-level features from the data for defect detection.

With the development of Graphics Processing Unit (GPU), deep learning technology has been broadly applied to various real-world applications^4,5^. By utilizing the benefits of GPUs, most deep learning networks have also been applied to on-fly detection across all aspects of industry^6,7^. These networks strongly promote the development of industrial inspection and address the disadvantages of classical detection technology. For example, Shifted Windows (SWIN) Transformer^8^, in which hierarchical feature maps are built by merging image patches in deeper layers, has linear computation complexity to input the image size and is used to resolve the deficiencies of Convolution Neural Network (CNN) on feature extraction. A shift operation is used to improve the receptive field of CNN. An improved Single Shot MultiBox Detector (SSD) algorithm with a region of interest^9^ was proposed to detect the defects of filling line containers^9^. This work indicates that the background noise can be suppressed in the region of interest. Although the performance of deep learning networks exceeds human performance in some specific domains, there still exists difficulty in detecting defects, especially in very high-resolution images. This is caused by the following two reasons. First, in practice, the shape and size of surface defects of industrial products are different; using an image algorithm for feature extraction requires many resources for algorithm design, which shows that its universality for the target object is poor. Second, in high resolution images, compared with regular objects, small objects have less information and the training of small objects is difficult to mark. This leads to poor performance when directly employing the previous object detection method for small object detection. Moreover, the detect methods designed for small objects are often too complex or specific. For example, for small targets such as bottle in PASCAL VOC dataset^10^, the features extracted from deep network contain little information about the small target^11,12^. Therefore, detecting small and various defect requires a well-designed feature learning network.

In this paper, motivated by two recent studies^9,8^, we establish a cylinder liner defect database and propose a Transformer network with Block division approach and Mask mechanism (TBM) to detect cylinder liner surface defect. More specifically, the defect patches are first collected as a training set. Then, with these training data, the Swin transformer, which is an encoder-decoder that is used as the backbone for feature extraction of the mask-RCNN network, learns the attention region. For the testing procedure, a mask mechanism that is based on morphology and used to extract the region of interest is proposed, and the Swin transformer is applied to detect the defects in regions of interest.

The key contributions of this paper are described as follows:

- To the best of our knowledge, we provide the first publicly available cylinder liner defect database.
- We propose a new defect detection system based on transformer with a block division approach and a mask mechanism to address the small defect detection problem for cylinder liners.
- We compare several state-of-the-art algorithms for object detection on the cylinder liner database and provide the baseline results for further research. Additionally, the proposed method is demonstrated to obtain promising and considerable performance in cylinder liner defect detection.

The remainder of this paper is organized as follows. In Section “Related work”, literature closely related to our proposed method is presented. In Section “Cylinder liner defect database”, our cylinder liner defect database is described. In Section “System architecture”, the proposed method for defect detection is presented. In Section “Experimental results and discussion”, the experimental results on the cylinder liner defect database are shown and discussed. In Section “Conclusion”, this paper is concluded.

## Related work

### Object detection algorithm

The feature extraction ability of deep learning is better than that of artificially designed feature extraction operator, so using CNNs for defect detection has become a research hotspot in the field of contemporary object detection. Currently, object detection algorithms based on CNNs can be divided into two categories: one-stage and two-stage object detection algorithms.

#### One-stage object detection algorithms

One-stage object detection algorithms are utilized to directly predict object bounding boxes for an image in a one-stage fashion. In other words, there are no intermediate tasks that must be performed to output the product. The most common examples of one-stage object detectors are SSD^13^ and You Only Look Once v3 (YoloV3)^14^. The SSD^13^ is a single-stage object detection method that discretizes the output space of bounding boxes into a set of default boxes over different aspect ratios and scales per feature map location. SSD’s architecture builds on the venerable VGG-16 architecture but discards the fully connected layers. Instead, a set of auxiliary convolutional layers are added, thus enabling the extraction of features at multiple scales and progressively decreasing the size of the input to each subsequent layer. Additionally, the successful SSD method utilizes the proposed multibox loss function, combining condition loss and location loss. This algorithm successfully integrates regression and classification tasks into the overall CNN structure and obtains the target category information and location information directly through a convolutional neural network. Under the action of the anchor mechanism, the region recommendation algorithm is cancelled. These two improvements greatly improve the detection efficiency of the network. In YoloV3^14^ model, DarkNet-53 backbone is adopted. The backbone network improves the feature extraction ability of the model. At the same time, under the ideas of dense convolutional network (DenseNet) and feature pyramid network (FPN), the model can detect small targets more accurately, the feature pyramid is formed, and the fusion between features is realized, which expands the semantic information of the low feature level. Moreover, the YoloV3 algorithm improves the loss function. In the category loss part, logic regression is used to replace the softmax function. Although the one-stage detection algorithm can quickly detect objects, they suffer from the class imbalance problem, more postprocessing and low accuracy rates.

#### Two-stage object detection algorithms

Two-stage object detection algorithms consider the detection task into two stages. In the first stage, the candidate region proposals are determined, and in the second stage, these proposals, which are generated from the regional proposal network (RPN) layer by using CNN, are classified. With the RPN network, which is a full convolution model, the extraction efficiency of candidate boxes is greatly improved. In the process of using the RPN network, an anchor mechanism and a nonmaximum suppression algorithm are used. The representative algorithms are R-CNN^15^, Fast R-CNN^16^, and Faster R-CNN^17^. R-CNN^15^ uses a selective search algorithm to generate candidate regions and then uses an image processing algorithm to scale the candidate regions to a fixed size. The processed regions are input into the designed CNN network for feature extraction, and the region classification is completed under the effect of the SVM classifier. Meanwhile, the fine-tuning of the border is completed, and the target information is finally obtained. Although the accuracy of this algorithm is high, considerable computing time is required to generate candidate regions. Meanwhile, when the image processing algorithm is used to fix the size of the region, there is also the problem of image distortion, which leads to the confusion of information. In addition, a large number of candidate regions show the problem of computational redundancy in CNN. To solve the problem of information loss caused by solidifying the size of candidate regions, Fast R-CNN^16^ is used to improve R-CNN. Different from R-CNN, Fast R-CNN inputs the whole image into CNN for calculation, and under the effect of ROI pooling, the output of CNN is fixed to a certain size of eigenvector. In this model, classification and regression are implemented in different networks, so although the defects are high, the detection speed is low. This model does not solve the problem that considerable computation time is required to generate candidate regions. Fast R-CNN also increases the computation of the model to a certain extent. To solve this problem, Faster R-CNN^17^ was proposed. Under the effect of ROI pooling and the corresponding hardware conditions, this model can accept any size of the input image. Here, the backbone network is designed to extract the features of the input image to obtain the corresponding feature map, which is shared by the RPN and the fully connection layer of the surface, reducing the amount of calculation to a certain extent. To solve the time problem of generating candidate regions, an RPN network is designed to generate the candidate regions. Compared with a one-stage network, the performance of Faster R-CNN is better than that of a one-stage network, but it is slower than a one-stage network.

### Defect detection algorithm

Given its development, deep learning has been widely applied in various defect detection tasks. Deep learning can be divided into two categories.

#### One-stage defect detection algorithms

Based on the advantage of the one-stage object detection algorithm, it is more efficient and elegant in design. For example, Chen et al. proposed using a generative adversarial network (GAN)^18^ and YoloV3 algorithm^14^ to detect defects in wafer die pie^6^. The pseudo defective images generated by GAN from the real defective images were used as the training image set. Then defects were measured based on the bounding boxes predicted by YoloV3. Although the detection speed is fast when using YoloV3, the major disadvantage is that it is difficult to guarantee that GAN can output more natural and realistic images, as GAN has more hyperparameters and is influenced by the background complexity and the size of the input images. Yin et al. proposed a real-time automated defect detection system for sewer lines by using a deep-learning algorithm^19^. They used YoloV3, which was trained with a dataset of 4,056 samples with six types of defects and one type of construction feature. Although it achieved a mean average precision of 85.37%, the system was influenced by the noise of the background. An improved SSD algorithm was proposed to detect the surface defects of filling line containers^9^. More specifically, the VGG-16 network was replaced by MobileNet, which strongly simplifies the detection model and increases the recognition rate to 95%. Before feeding an image into SSD, Hough circle detection was used in the preprocessing phase to locate the edge of the cover and mitigate the impact of useless background on the recognition accuracy. Compared with YoloV3^19^, VGG-16 segmented the region of interest, which suppressed the background noise and improved the detection rate of very small defects. This indicates that according to specific defects, the segmentation can improve the performance.

#### Two-stage defect detection algorithm

Based on the advantage of the one-stage object detection algorithm, the two-stage detectors have superior accuracy. To date, some works have been presented to detect defects in various fields^20–23^. For example, Perez et al. first studied defect detection using convolution neural network (CNN)^20^. As mold, deterioration, and stains frequently occur on the surface of buildings, a pretrained CNN classifier with VGG-16^24^ as the backbone was proposed, and finally CNN was combined with class activation maps for target location. In their work, this model considers an image to belong to only one category. This means that multiple types of defects are not considered for the VGG architecture. Therefore, this architecture may not be suitable for more than binary classes. Duong and Kim implemented a deep neural network for bearing fault diagnosis^22^. They segmented the continuous signal into lengths of 500 and 1000 data points for bearing fault diagnosis. Kumar et al. proposed an ensemble of binary CNNs for automated defect detection based on CCTV inspection of sewers^21^. However, their method^21,22^ required many labeled fault samples to train the fault detection model.

Considering the small number of defective components, Gibert et al. proposed deep multitask learning by combining multiple detectors to learn a robust anomaly detector, which resolves the problem caused by the number of different possible failure modes^25^. The multiple detectors contain four different tasks that include the detection of the missing, damaged and good fasteners, the binary classifications of good and bad fasteners, and the classification of good fasteners. Tabernik et al. presented a segmentation-based deep learning architecture for the detection and segmentation of surface anomalies^7^. More importantly, their proposed method enables the model to be trained using a small number of samples. The architecture was formulated as a two-stage design. In the first stage, a segmentation network was implemented to perform pixelwise localization of the surface defect. The benefit of this approach is to increase the effective number of training samples and prevent overfitting by effectively considering each pixel as an individual training sample, However, only 25-30 defective training images are able to be learned. In the second stage, an additional network for binary classification was built on top of the segmentation network. However, acquiring pixel-level labels is both time- and labor-intensive. Di et al. proposed a semisupervised surface defect detection method based on GAN for hot-rolled strip steel workpieces^26^. In their method, they used a convolution encode-decode module for unsupervised feature learning and trained an autoencoding module with one classification layer as a GAN discriminator. Both labeled and unlabeled samples have been used to train classifiers with different learning strategies but have been unable to predict defect regions.

## Cylinder liner defect database

**Figure 1:**
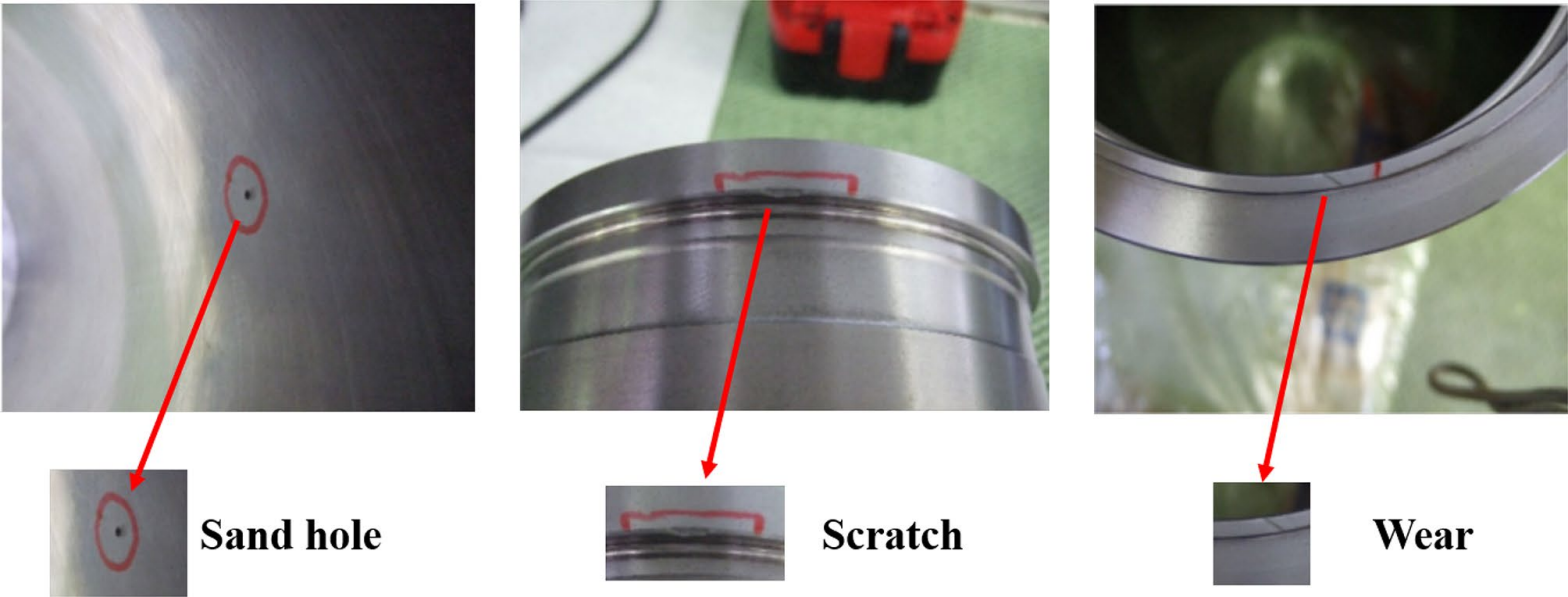
Three frequently occurring cylinder liner defects. The images from left to right represent sand hole, scratches, and wear, respectively.

To the best of our knowledge, there is currently no database of cylinder liner defects. Therefore, we collect a new cylinder liner defect database from industry, namely, the Cylinder Liner Defect (CLD) database. In general, there are several types of defects on cylinder liners, such as sand holes, bumps, cracks, oil stains, scratches, and wear. During the manufacturing process of cylinder liners, various defects, such as sand holes, crack, and wear, may occur due to the uneven stress distribution, temperature, and impurities. In this paper, we focus on three frequently occurring defects, including sand holes, scratches, and wear, which are described as follows:

- Sand hole: This is mainly caused by the casting production form of the cylinder liner. The gas and nonmetallic inclusions cannot be discharged from the liquid metal before solidification, resulting in sand hole defects on the cylinder liner^27^. As sand holes easily deteriorate the performance of cylinder liners, even causing cylinder collapse and water leakage, they are the primary defect of cylinder liners. An example is shown in Figure 1a. As seen from Figure 1a, the sand hole is very small.
- Scratch: During casting of the cylinder liner, once the actual deformation caused by the combined action of various stresses exceeds its plastic limit, it will result in cracks on the cylinder liner^28^. These cracks affect the service life and replacement cycle of the cylinder liner, which undoubtedly leads to potential safety hazards in the service stage of the cylinder liner. A scratch defect is shown in Figure 1b. The defect appears as a snowflake crack.
- Wear: This often occurs in the process of production and transportation. In the production process, the generated waste materials will damage the cylinder liner, and in the transporting process, friction and collision will produce massive wear defects. For an internal combustion engine, wear in cylinder liner damages the tightness of the cylinder liner and also degrades the power of the engine. The wear defect is shown in Figure 1c. There is a line-type wear along the edge of the cylinder liner.

**Figure 2:**
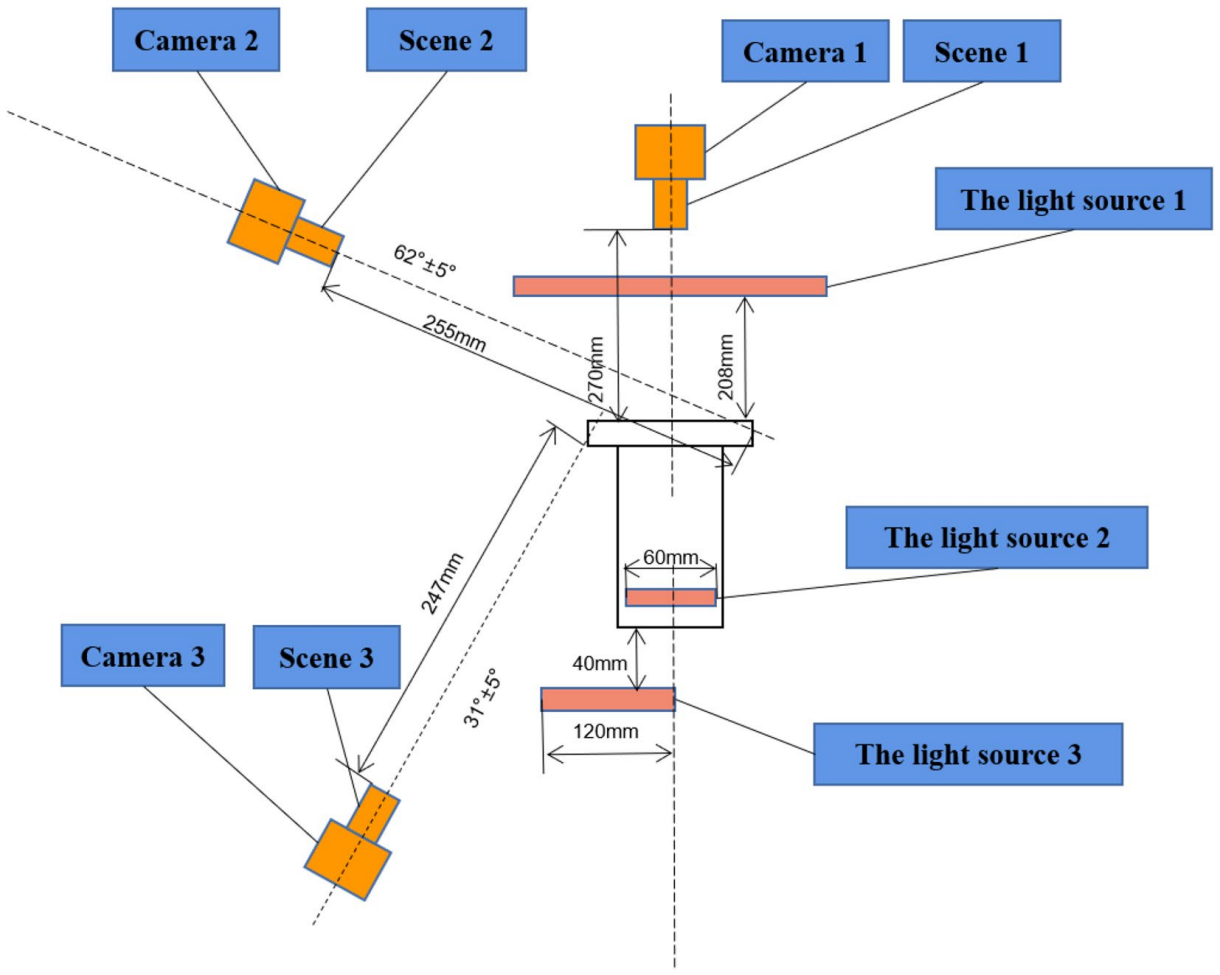
The structure of data collection for cylinder liner defects. Best viewed in color.

**Figure 3:**
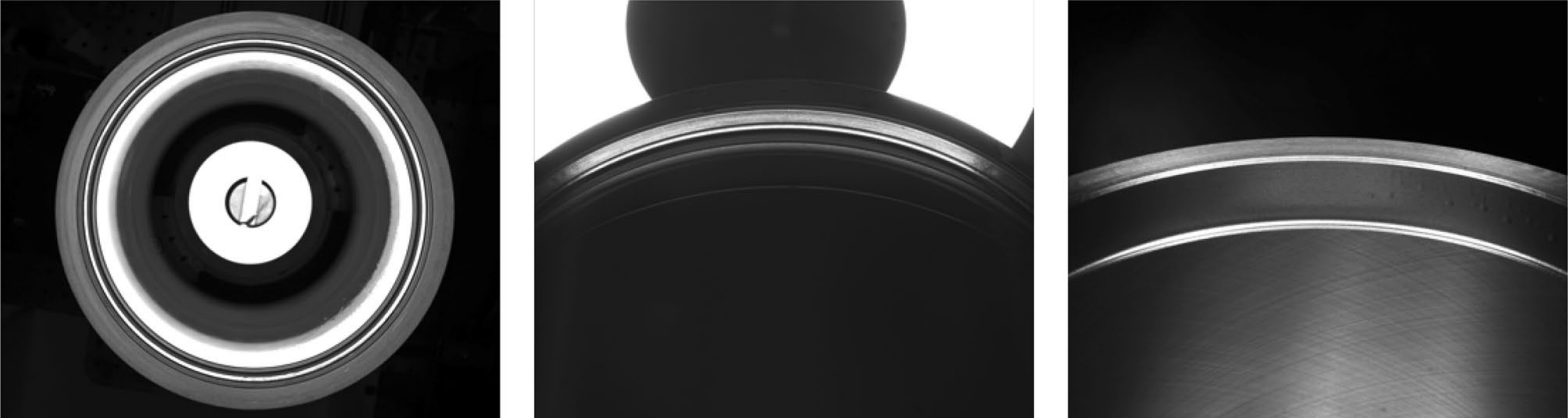
Three cylinder liner examples taken by an area-array CCD camera.

**Figure 4:**
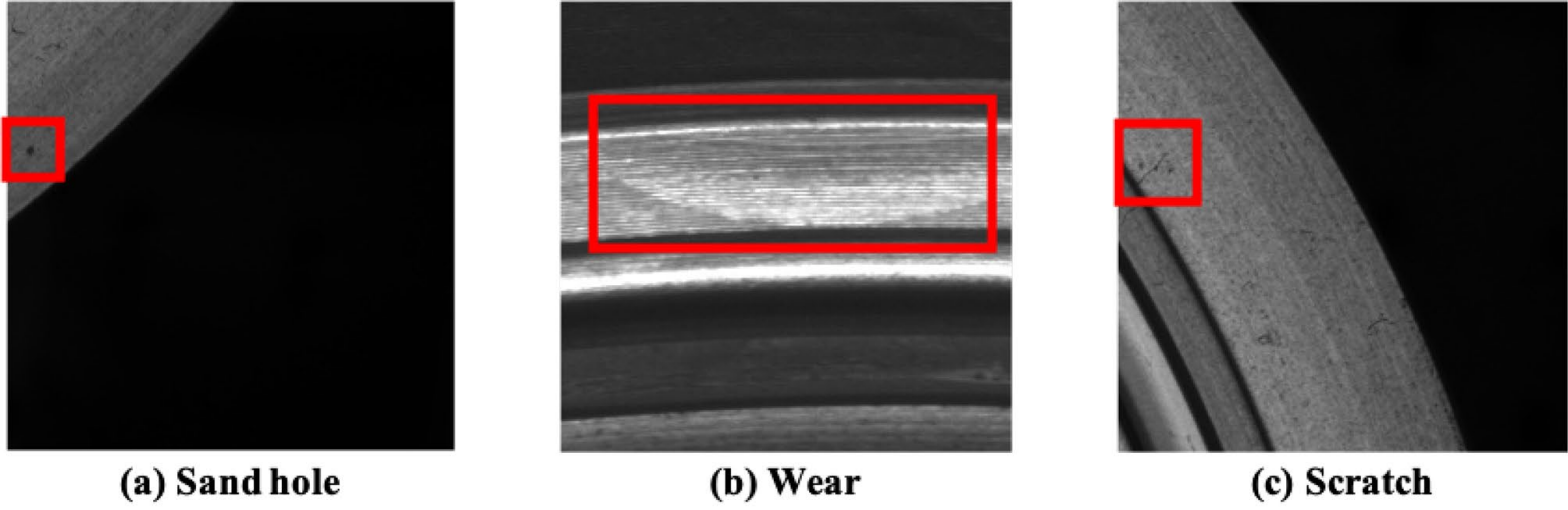
The local enlarged region of the cylinder liner, where the defect is located inside the red rectangle.

Following the existing utilized methods ^29,30^ in industrial data collection, we use three charge-coupled device (CCD) industrial cameras for image acquisition and three light emitting diodes (LEDs) as the light source, the positions of which are illustrated in Fig. 2. More specifically, considering the defects existed different positions of the cylinder liner, we use three area-array CCD cameras (Camera 1, Camera 2, and Camera 3, as indicated in Fig. 2) to collect the defect images from the upper top surface (Scene 1), skirt (Scene 2), and inner wall (Scene 3) of the cylinder liner. The advantage of this process is to quickly and intuitively obtaining two-dimensional cylinder liner images. We collected a total of 7500 images, some of which are shown in Fig. 3. The defect annotation requires professional knowledge, and it also lacks of no specific standard for human annotation. Therefore, we asked two experts to manually annotate the surface defects. With two experts’ annotations, only 585 images with a size of $2448\times 2048$ contain defects, including sand holes, scratches, and wear. Three local enlarged defects are illustrated in Fig. 4.

## System architecture

**Figure 5:**
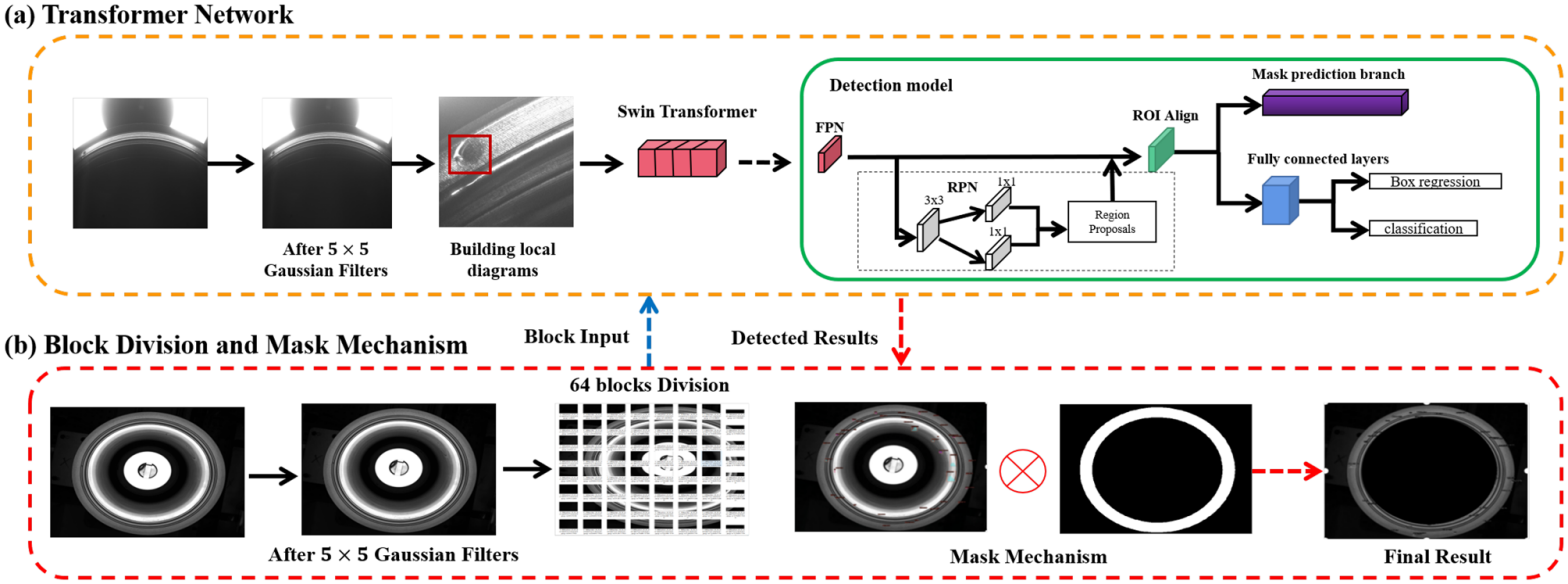
Our proposed framework for detecting very small defects on high-resolution cylinder liner images. Best viewed in color.

Our proposed framework is shown in Fig. 5. It consists of two stages: a transformer network stage and a block division and mask mechanism stage. The transformer network focuses on training a deep network based on a local defect dataset, in which each image contains one defect, while the block division method separates an image into 64 blocks and then uses the network to detect each block, subsequently mapping the detected results into an original image, and the mask mechanism suppresses the noise in the background. In this section, we will detail each stage.

### Transformer network

The Swin transformer^8^ is capable of serving a general-purpose backbone for computer vision. It uses a hierarchical architecture to address problems such as the scale of visual entities and the high resolution of pixels in images. To enhance the performance of defect detection of cylinder liners, we propose using a Swin transformer network followed by a detection model. To improve the accuracy of the Swin transformer, we propose an image processing method to enhance the image quality. Here we use a Gaussian filter, which is a linear smooth filter, to primarily remove the Gaussian noise.

#### Swin transformer

**Figure 6:**
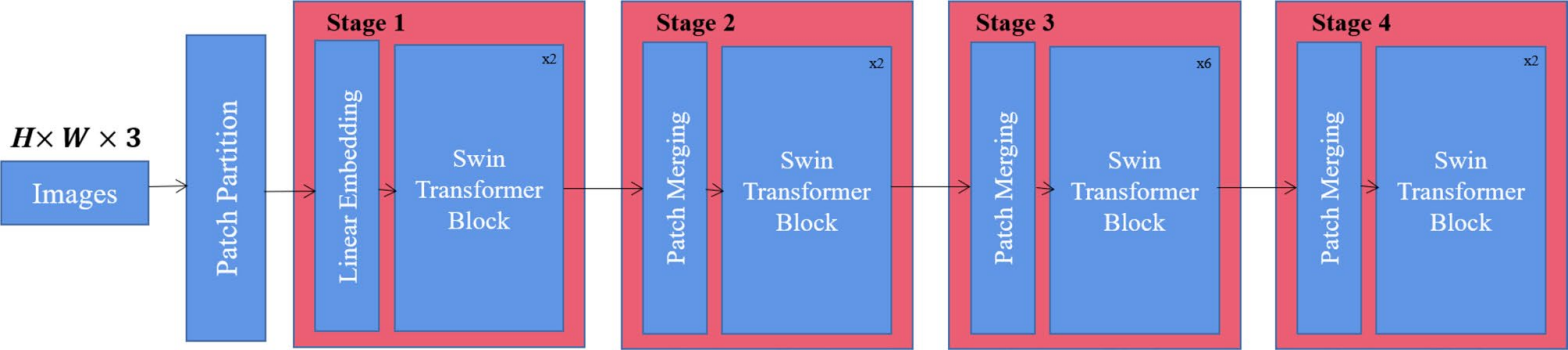
Swin transformer architecture^8^ used in our paper.

The Swin transformer is one variation of an encoder-decoder architecture. Figure 6 shows its architecture, where *H* and *W* are the height and width of an image, respectively. Following^8^, we briefly describe the Swin architecture by borrowing their some formulations.

As shown in Fig. 6, the Swin transformer mainly uses three modules to build its architecture. It consists of the patch partition module, linear embedding module, and Swin transformer block. The patch partition module splits an input RGB image into nonoverlapping patches, where the feature of each patch is a concatenation of the raw pixel RGB value, while the linear embedding module predicts each patch to any arbitrary dimension *C* on this raw-valued feature. A Swin transformer block provides efficiency by limiting self-attention computation to nonoverlapping local windows. For the Swin transformer, the key component is the Swin transformer block.

##### Swin transformer block

**Figure 7:**
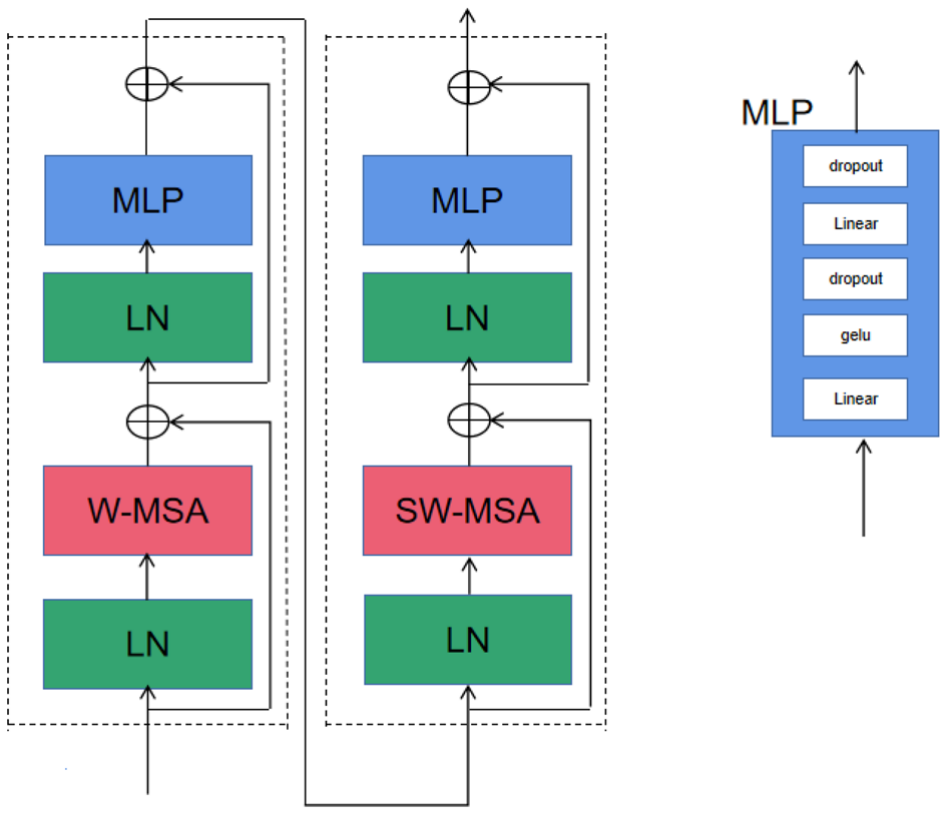
Block of swin transformer^8^ used in our paper, where LN is layer normalization layer.

This block is mainly composed of a window based self-attention layer (W-MSA) module and a shift window based multihead self-attention layer (SW-MSA) module. The Swin transformer block is shown in Fig. 7. In Fig. 7, W-MSA is used to calculate window based attention, while SW-MSA attention after sliding the window. Layer normalization (LN) is used to normalize the data of the input network, and MLP is composed of two fully connected layers, which successively pass through the fully connected layer, GELU activation function, dropout layer, full connected layer, and dropout layer. Using W-MSA and SW-MSA, consecutive Swin transformer blocks are computed as,

1 $$\begin{aligned} z^{k+1}&=\text {MLP}(\text {LN}(\text {W-MSA}(\text {LN}(\hat{z}^{k}))+\hat{z}^{k}))+\text {W-MSA}(\text {LN}(\hat{z}^{k}))+\hat{z}^{k}, \end{aligned}$$

2 $$\begin{aligned} \hat{z}^{k+1}&=\text {MLP}(\text {LN}(\text {SW-MSA}(\text {LN}(z^{k+1}))+z^{k+1}))+\text {SW-MSA}(\text {LN}(z^{k+1}))+z^{k+1}, \end{aligned}$$

where $\hat{z}^{k}$ denotes the output features of the *k*th block, where $k=1,\ldots ,4$ in our paper.

##### Pipeline

The patch partition module splits an input RGB image into nonoverlapping patches. Then, the linear embedding module and Swin transformer block are composed. The pipeline of the linear embedding and Swin transformer block is referred to as ”Stage 1“. To produce a hierarchical representation, three additional stages are applied. Importantly, the Swin transformer uses patch merging layers to reduce the number of patches, when the network becomes deeper. More specifically, in ”Stage 2“, the features of each group of $2\times 2$ neighboring patches are concatenated by using the first patch merging layer, and then a linear layer is applied on the $4C-$dimensional concatenated features. This results in the number of patches decreasing by a multiple of 4 and the output dimension as 2*C*. Swin transformer blocks are applied afterward for feature transformation, with the resolution maintained at $\frac{H}{8}\times \frac{W}{8}$. This procedure is repeated twice, as ”Stage 3“ and ”Stage 4“, with output resolutions of $\frac{H}{16}\times \frac{W}{16}$ and $\frac{H}{32}\times \frac{W}{32}$, respectively.

##### Discussion

The characteristics of the Swin transformer are as follows: (1) The Swin transformer formulates a hierarchical feature map by merging the patterns across deep levels, and has also linear computational complexity, because only self attention calculations are performed for each local window. Among them, windows are not overlapped, and the number of patches in each window is fixed. (2) The shifted window spans the upper layer and improves the performance of the model. Query patches in the same window share the same key set, which improves the efficiency of accessing memory.

#### Detection model

The detection model is primarily based on the basic architecture of Mask RCNN^31^. The pipeline is realized by using feature pyramid networks (FPN), followed by region proposal network (RPN), fully concatenated layers, and a binary mask prediction branch, as depicted in Figure 5. Nonmaximum suppression (NMS) is performed as a postprocessing step to obtain the final set for detection. In the following section, we briefly introduce how the detection model is realized. More technical details can be found in the Mask RCNN study^31^.

In our approach, we implement ResNet50 as the backbone for FPN to extract defect image features. FPN aims at building high-level semantic feature maps at all scales. It takes a defect images and exports a five-scale feature pyramid, by using a top-down architecture. Then, according to the anchors, RPN with $3\times 3$ convolution and two $1\times 1$ convolutions is used to propose candidate object bounding boxes (that is, region proposals) in the different scales. Subsequently, features of region proposal are extracted by the RoI align layer, which removes the harsh quantization of RoIPool, properly aligning the extracted features with the input. Then the features are fed into the fully connected layers and softmax layers, which can finally estimate softmax probability of defects (i.e.,  ’classification‘ in Fig. 5) and also refine the bounding box positions (i.e., ’Box regression‘ in Fig. 5)for the defect targets. At the same time, the features are fed into the mask prediction branch, which consists of four convolution layers and one deconvolution layer, to predict the defect target mask.

### Block division and mask mechanism

In the real-world implementations, we expect the Swin transformer to better detect small defects in the high-resolution images. However, according to our empirical research, it is very difficult to locate the defect, once the high-resolution image is used as the network input. This is because the image scale will make the defect be missed. Therefore, in order to quickly and accurately detect the defect, we propose block division (BD) method for the detection procedure. More specifically, we divide the high-resolution image ($2448\times 2048$) into 64 blocks, and then use the trained Swin transformer to detect each block. Finally, we map these detected results into the high-resolution image. Our proposed approach can be used to improve the detection precision. Our experiments solidly validate this proposal.

Although the block division method can be used to resolve the small defects existing in high-resolution images, for cylinder liners, there are some special characteristics, such as the useless region. For example, in the first example of Fig. 3, the background is outside the outlier cycle and inside the inner cycle. These useless regions will cause the noise in the detection performance. Therefore, we propose a mask mechanism to address these issues. The mask mechanism is implemented to design the specific binary mask, where ‘1’ means foreground and ‘0’ means background. With the specific mask, the region of interest will be found. The advantage is to suppress the noise due to background. In this implementation, we multiply the detected results by the mask so that the falsely detected results in the background will be removed. Considering the acquisition of cylinder liner images acquired from different cameras, we design three kinds of masks as follows:

- For the rough region made from the upper camera (as shown in the first image of Fig. 3), we set the appropriate threshold for image binarization. Then, we use morphological operations and closed operations to remove the pixels with small binarization and ensure that the binarization region contains the region of interest.
- For the upper end face image (as shown in the second image of Fig. 3), we measure the coordinates of the upper end face image. According to the detected area in the upper face, we determine the center of the circle and radius of the maximum circle and the minimum circle. Last, we take a ring binary image as the region of interest of the upper end face image.
- There is another type of picture (as shown in the third image of Fig. 3). We manually design a specific mask to extract the region of interest.

**Figure 8:**
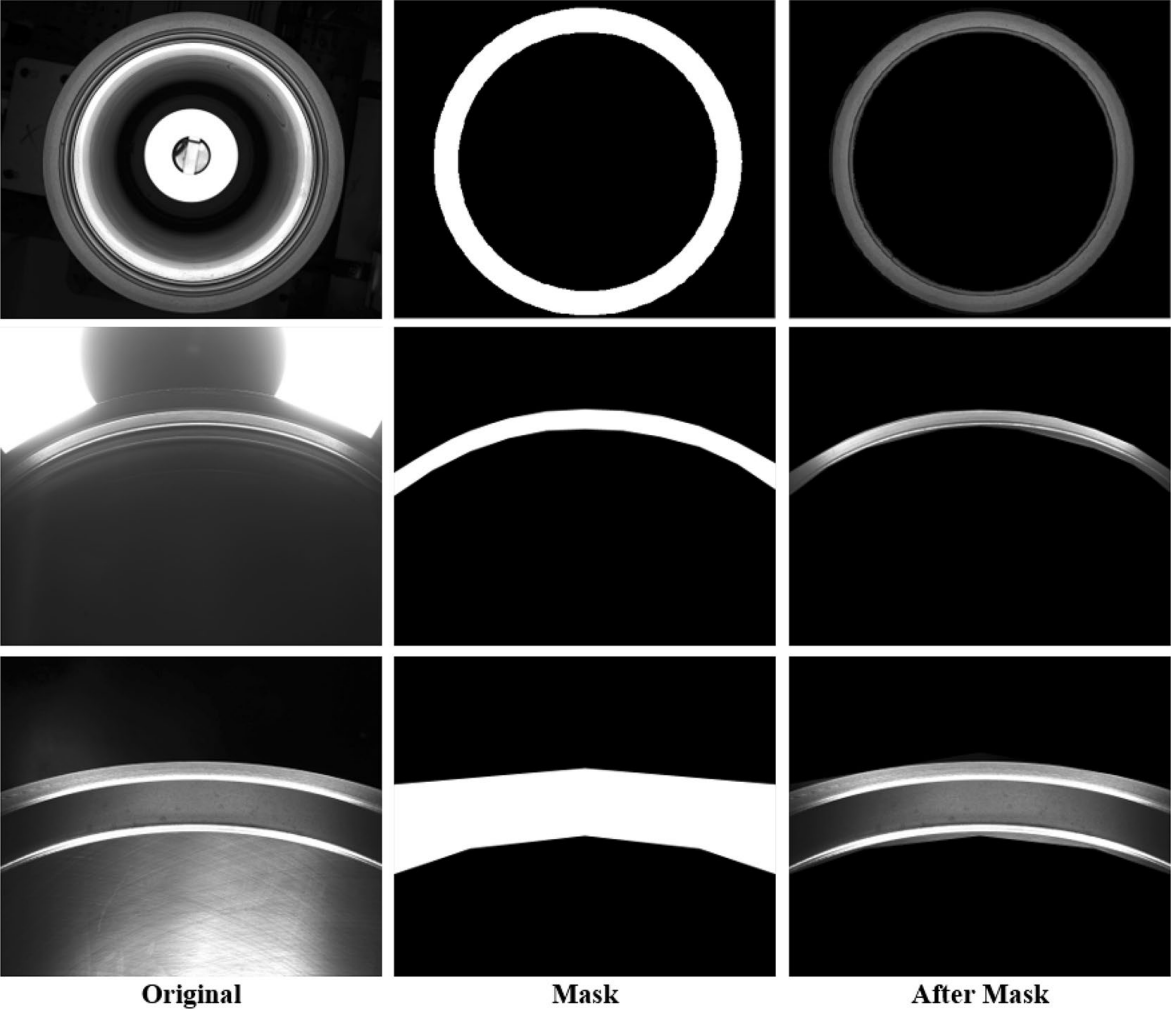
Our proposed mask mechanism for cylinder liner.

The masks for the three types of images are shown in Fig. 8.

## Experimental results and discussion

### Experiment setup

We conduct the experiments on our collected CLD database. For training the backbone network, we create the local surface defect dataset derived from CLD database. For the defect patch image, we extract it by using annotation information of the original cylinder liner surface defect. All defect patch images are normalized to a size of $256\times 256$. Each defect patch image only contains one defect. Last, we manually extract 1061 defect patch images from the training set as the ‘training defect subset’, and 118 images from the test set as the ‘test defect subset’. In the network training, number of epochs is set as 300, batch size as 6, learning rate as 0.0001, and decay value as 0.05. All codes are run on NVIDIA Titan RTX (24GB) and implemented based on Python 3.7 and PyTorch 1.8. The experiment settings are listed in Table 1.

**Table 1:** Experimental setting used in the training process.

Hyperparameter	Value
Training epochs	300
Batch size	6
Learning rate	0.0001
Weight decay	0.0.05

To evaluate block division and mask mechanism, we select 54 original high-resolution images as the test data. These data are used to verify the final performance of the proposed method.

In the prediction stage, we adopt the mean average precision (mAP), which is the mean precision over all classes as follows,

3 $$\begin{aligned} MAP = \frac{1}{n}\sum _{i=1}^{n}AP_{i} \end{aligned}$$

where *n* is the number of classes. $AP_{i}$ is the average precision of the *i*th class. For the *i*th class, we compute the precision and recall for this class at different class confidence thresholds (from 0.0 to 1.0).

4 $$\begin{aligned} AP_i = \int _{0}^{1}p_ir_idr_i, \end{aligned}$$

where $p_i = \frac{TP}{TP+FP}$, $r_i = \frac{TP}{TP+FN}$, *TP* is the true positive, *FP* is the false positive, and *FN* is the false negative.

### Ablation study

#### Backbone selection

**Figure 9:**
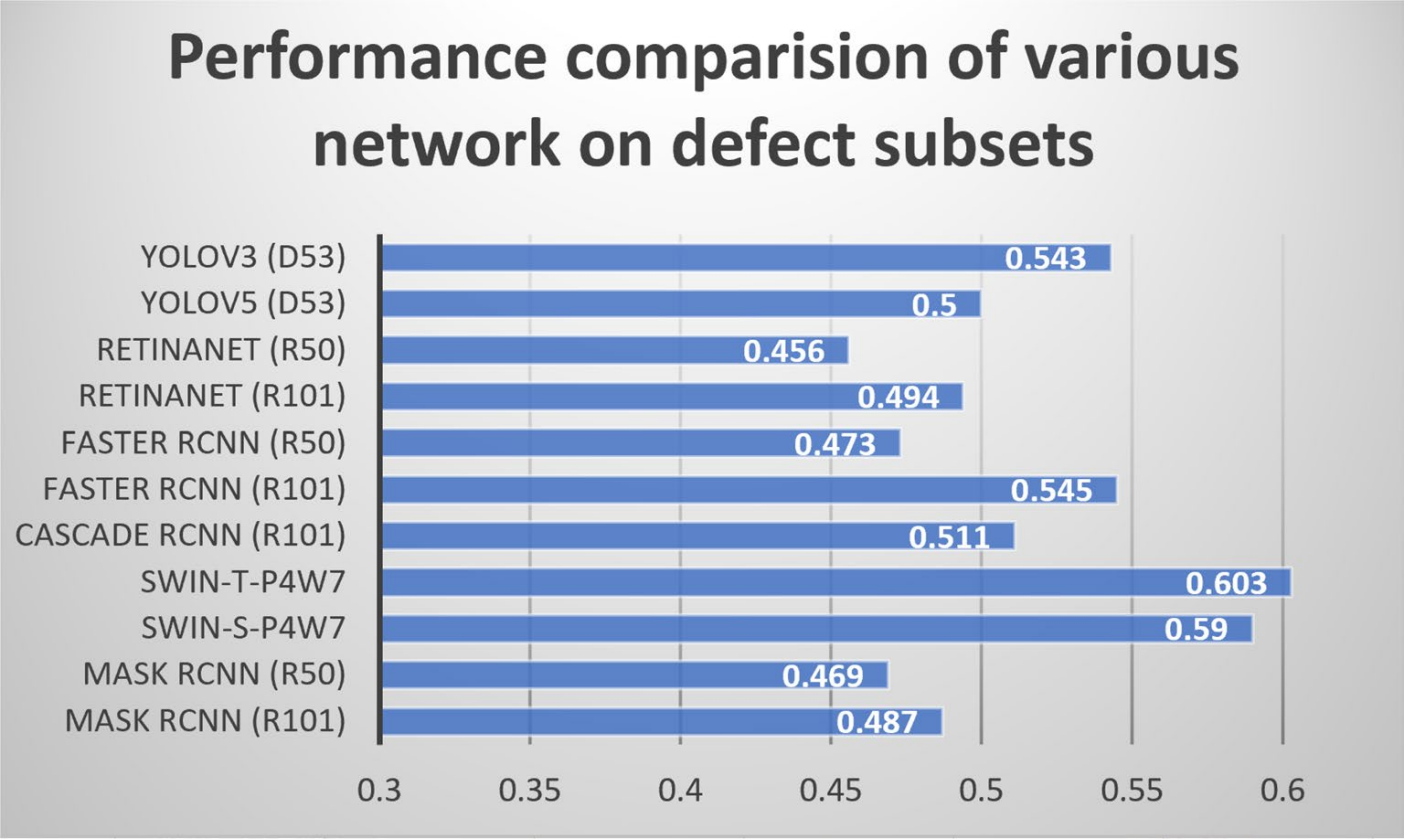
Performance comparison of various networks on the local defect database in terms of mAP. D53, R50, and R101 represent DarkNet53, ResNet50, and ResNet 101 backbones, respectively. SWIN-T-P4W7 and SWIN-S-P4W7 indicate very small Swin transformer models, respectively.

To validate the rationale of our proposed network, we compare our basic network with YoloV3^14^, YoloV5 (https://pjreddie.com/), RetinaNet^32^, Faster R-CNN^17^, Mask RCNN^31^, and Cascade RCNN^33^. The algorithm comparison is conducted on the local surface defect dataset. The performance comparison is shown in Fig. 9. Swin transformer outperforms the other state-of-the-art methods, especially using a very small model for Swin Transformer. A mAP of 0.603 is achieved using Swin transformer with a very small model; this result represents an increases in the mAP of 0.134 and 0.115 compared with the Mask RCNN with the Resnet50 and Resnet101 backbones, respectively. Considering the comparison, we choose the Swin transformer as our network.

#### Number of stages

To evaluate the influence of the stages, we study the results under the different numbers of stages. As shown in Fig. 6, the Swin transformer architecture has four stages, and a mAP of 0.706 is achieved. When we reduce the number of stages to one, which means that only ‘Stage 1’ is included, the mAP is 0.296. With more stacked stages, the mAP is 0.388, 0.57, and 0.6 for the two-stage, three-stage, and five-stage models, respectively. This indicates that the performance is improved with an increasing number of stages. However, when we use 5 stages, the performance is degraded. This may be explained by the fact that more than 4 stages will suppress the discriminative feature as the output resolution will be very small.

#### Evaluation of Gaussian filter

In our method, we use Gaussian filter as data augmentation. To see the influence of the Gaussian filter, we compare the size of the Gaussian filter, including $3\times 3$, $5\times 5$, and $7\times 7$. The comparison is shown in Table 2. It is seen that the Gaussian filter with $5\times 5$ slightly outperforms that with $3\times 3$ filter. Moreover, we further investigate the mAP of the three categories. The results are shown in Table 3. It is seen that using a Gaussian filter with $5\times 5$ improves the precision of detection on the wear class, by increasing the mAP of 0.065, compared with that with $3\times 3$ filter. Unfortunately, the Gaussian filter with $5\times 5$ fails to considerably improve the performance of the sand hole class, but it is still competitive with the Gaussian filter with $3\times 3$ on scratch class. Considering the abovementioned analysis, we choose $5\times 5$ for the Gaussian filter.

**Table 2:** Performance of the Swin transformer under various Gaussian filter parameters.

Method	Size	mAP
Without Gaussian filter	$-$	0.671
With Gaussian filter	$3\times 3$	0.700
With Gaussian filter	$5\times 5$	**0.706**
With Gaussian filter	$7\times 7$	0.649

The best result is in bold.

**Table 3:** Performance of Swin Transformer with Gaussian filters on three classes, where we compare the results under the filter sizes of $3\times 3$ and $5\times 5$.

Gaussian filter	Wear	Scratch	Sand hole	mAP
$3\times 3$	0.613	**0.755**	**0.733**	0.700
$5\times 5$	**0.678**	0.749	0.692	**0.706**

The best result is in bold.

**Table 4:** Ablation study of the TBM, where S means that we use the local defect dataset to train the network, BD represents the block division method, and the best result is in bold.

Method	Module			Class			mAP
	S	BD	Mask	Wear	Scratch	Sand hole	
Original method				0.286	0.061	0.041	0.129
TBM	$\checkmark$			0.002	0.02	0.001	0.008
TBM	$\checkmark$	$\checkmark$		0.344	0.174	0.314	0.277
TBM	$\checkmark$	$\checkmark$	$\checkmark$	0.628	0.455	0.529	**0**.**537**

#### Evaluation of block division

This experiment aims to evaluate the performance of the block division method. Here, we compare them with the “Original method”. In the original method, we use the original high-resolution image with its corresponding defect labels for training and testing. The comparison results are reported in Table 4. The “Original method” obtained the lowest mAP of 0.129 among all methods. Additionally, for all classes, it loses the capability to detect very small defects. This can be explained by that the feature pyramid method causing the small defect to disappear with the deeper layer. In contrast, the block division method focuses on the several very small regions, such that FPN will not have a negative influence on the detection. To some extent, the block division method increases all the classes. For example, for the sand hole class, the block division method increases the detection precision by 0.273. The comparison demonstrates that the block division method can better address the abovementioned problem.

#### Evaluation of mask mechanism

**Figure 10:**
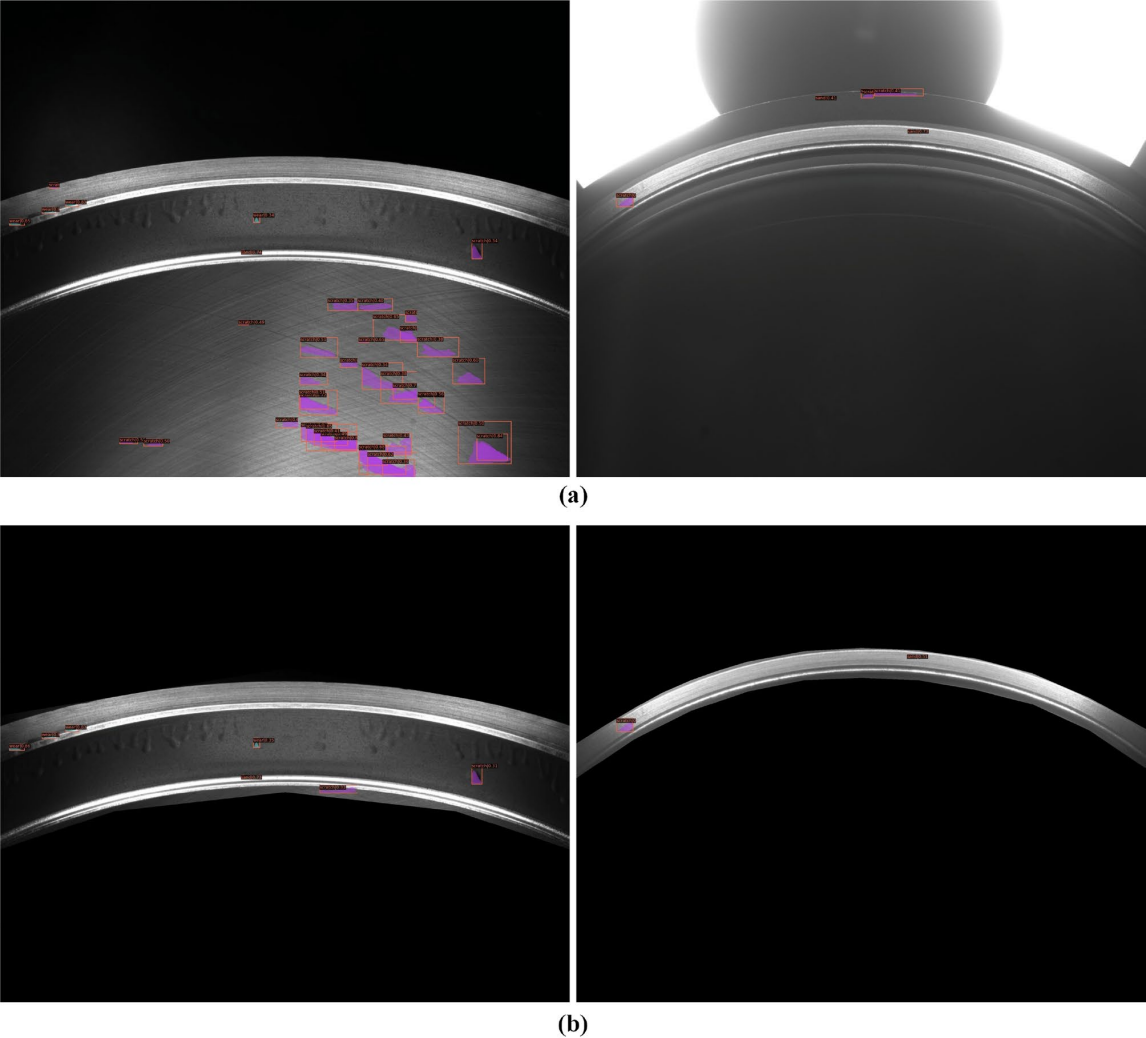
Visual analysis of two detected results (**a**) before and (**b**) after adding the mask mechanism. Best viewed in zoom and color.

We evaluate the TBM without/with the mask mechanism to determine its contribution to detection. The quantitative analysis is shown in Table 4. It is seen that with the mask mechanism, the block division method is increased from 0.277 to 0.537 by increasing the mAP to 0.26. The mAPs of wear, scratches and sand hole are increased by 0.284, 0.281, and 0.215, respectively. These results demonstrate that adding the mask mechanism promisingly improves the performance of the block division method. Moreover, we qualitatively analyze the mask mechanism in Fig. 10. In Fig. 10b, we can see that for the first example (in the first column), there are too many falsely detected results in the background. In fact, this background does not contain information about the cylinder liner. The mask mechanism removes the detected errors and increases the performance of the block division method. The influence is the same as in the second example (in the second column). Therefore, the mask mechanism can suppress the influence of the background in our proposed method.

### Performance comparison

**Table 5:** Parameter setup of our proposed method.

Component	Parameter
Gaussian filter	$5\times 5$
Number of blocks	64
Number of stages	4

To evaluate the performance of our proposed method and the existing object detection for cylinder liner surface defect detection, we use five deep learning networks for comparison. The parameters of our proposed method are shown in Table 5. The compared five deep learning networks include You Only Look Once (Yolo)V3^14^, YoloV5 (https://pjreddie.com/), RetinaNet^32^, Faster Region-CNN (RCNN)^17^, Mask RCNN^31^, and Cascade RCNN^33^. We used ResNet50 as backbone for Mask RCNN and Faster RCNN, and ResNet101 as backbone for Mask RCNN, Cascade RCNN, Faster RCNN, and RetinaNet. During training, we directly utilize the default parameters for these baseline networks. The results are presented in Table 6.

According to Table 6, among all baseline algorithms (w.o. TBM), YoloV5 achieves the best mAP of 0.16, in which accuracies of 0.309, 0.089, and 0.084 are obtained for the wear, scratch, and sand hole classes, respectively. For the wear class, the best result is obtained by Faster RCNN with ResNet 50. For the scratch class, the best accuracy of 0.121 is obtained using Cascade RCNN. For sand hole class, the best accuracy of 0.084 is obtained using YoloV5. Scratch and sand hole classes are difficult to detect. This may be explained by the fact that scratch and sand hole defects are very small. In contrast, a mAP of 0.537 and accuracies of 0.628, 0.455, and 0.529 for the wear, scratch, and sand hole classes, respectively, are obtained using our proposed TBM. Compared with YoloV5, our proposed method increases the mAP by 0.357. Additionally, for sand hole class, the increased accuracy is 0.445, and for scratch, the accuracy is increased by 0.366.

Based on block division and the mask mechanism, we compared the transformer architecture with other deep learning frameworks. The results are shown in the last 10 rows. As seen from the results, among all compared algorithms, Faster RCNN (R101) achieves the second-best mAP of 0.429. Instead, our proposed TBM with transformer architecture obtains a mAP of 0.537, and accuracies of 0.628, 0.455, and 0.529 for the wear, scratch, and sand hole classes, respectively. Compared with TBM with Faster RCNN (R101), the performance is increased by 0.128. It is seen that the TBM significantly improved the performance of defect detection.

In addition to performance, we compare our proposed method with other approaches in computing efficiency. As indicated in Table 6, without TBM, among Mask RCNN, Cascade RCNN, Faster RCNN, RetinaNet, YoloV3, and YoloV5, YoloV5 has the highest efficiency of 92.1 images/second, and Mask RCNN has the slowest detection speed. Even with TBM, YoloV5 achieves the fastest detect speed, in which the fps is 93.6 images/second. For most cases, the TBM increased the computing efficiency. For example, for the Mask RCNN, the detection speed is increased by approximately 4 images per second. In addition, even when using the transformer architecture, the computing efficiency is 21.4 images per second, which is still competitive with other methods. Therefore, the experimental results demonstrate that the TBM has better computing efficiency in defect detection.

### Discussion

As shown in Fig. 9, we use a local defect database, in which each image contains only one defect, to validate the Swin transformer, a mAP of 0.603 is achieved. The degradation in the detection accuracy is caused by two reasons: (1) each block may contain more than one various defect and (2) defects are diverse, when we use the TBM in the original high-dimension image.

**Table 6:** Performance comparison and detection efficiency between our proposed method and the state-of-the-art algorithms in object detection.

Method	Backbone	Class			FPS (image/s)	mAP
		Wear	Scratch	Sand hole		
Mask RCNN^31^	ResNet50	0.166	0.032	0.018	25.7	0.072
Mask RCNN^31^	ResNet101	0.288	0.057	0.078	16.7	0.141
Cascade RCNN^33^	ResNet101	0.254	0.121	0.068	16.5	0.148
Faster RCNN^17^	ResNet50	0.315	0.072	0.047	24.8	0.145
Faster RCNN^17^	ResNet101	0.302	0.081	0.078	19.4	0.154
RetinaNet^32^	ResNet50	0.163	0.052	0.054	26.1	0.09
RetinaNet^32^	ResNet101	0.167	0.110	0.03	20.1	0.102
YoloV3^14^	D53	0.241	0.009	0.007	84.3	0.086
YoloV5	D53	0.309	0.089	0.084	92.1	0.16
TBM	Mask RCNN (R50)	0.567	0.309	0.31	26.2	0.395
TBM	Mask RCNN (R101)	0.571	0.320	0.326	20.6	0.406
TBM	Cascade RCNN (R101)	0.511	0.352	0.373	17.1	0.412
TBM	Faster RCNN (R50)	0.429	0.372	0.4	27.6	0.4
TBM	Faster RCNN (R101)	0.473	0.376	0.437	21.4	0.429
TBM	RetinaNet (R50)	0.448	0.304	0.446	31.1	0.399
TBM	RetinaNet (R101)	0.502	0.332	0.374	23.6	0.402
TBM	YoloV3	0.517	0.351	0.378	85.5	0.415
TBM	YoloV5	0.51	0.343	0.358	93.6	0.404
**TBM**	**Transformer**	0.628	0.455	0.529	21.4	**0.537**

The best and second best results are in bold and underlined, respectively. R50 and R101 represent ResNet50 and ResNet101, respectively.

## Conclusion

In this paper, a transformer with a block division and mask mechanism (TBM) is proposed to considerably perform both defect detection and classification tasks on our new cylinder liner defect database for a cylinder liner defect against complex industrial scenarios. The proposed TBM can not only quickly detect very small cylinder liner defects but also suppress the noise caused by the background. The visual and quantitative experimental results have shown that our detection algorithm boosts the performance of the network under high-resolution images and provides a generic framework for other networks. The experimental analysis also established that the block division and mask mechanism can help transformers with greater accuracy. Experimental results have shown that the proposed method achieves an average of 0.537 mAP for three defects. In addition, the proposed method outperformed other state-of-the-art algorithms, including YoloV3, YoloV5, and Mask RCNN, showing 0.451, 0.377, and 0.396 improvements in accuracy, respectively.

In the future, we will study an approach to resolve the few-sample problem, as our database contains less than 1000 images. Additionally, the surface of an industrial cylinder liner is a three-dimensional curved surface. In our database, the cylinder liner defect image is captured by an area array camera, which causes the inconsistency in the background acquisition conditions in different areas, and then affects the detection results. We will design an appropriate image acquisition mechanism and select a linear array camera for image acquisition to improve the image quality.

## Data Availability

The datasets generated during and/or analysed during the current study are available from the corresponding author on reasonable request.
